# Long‐Term Trends and Projections of Multiple Myeloma Across Three Continents: A Comparative Study of China, the United States of America, the Russian Federation, England and France (1990–2036)

**DOI:** 10.1002/cam4.70999

**Published:** 2025-06-18

**Authors:** Huiqiang Wu, Zhiyin Cai, Wanyi Liu, Zechuan Wang, Baoying Qiu, Weihui Liu, Weihuang Zhuang

**Affiliations:** ^1^ The Second Affiliated Hospital of Fujian Medical University Quanzhou Fujian Province China; ^2^ The Second Clinical Medical College Fujian Medical University Fujian China; ^3^ Department of Hematology, The Second Affiliated Hospital Fujian Medical University Quanzhou Fujian Province China; ^4^ Department of Urology The Second Affiliated Hospital of Fujian Medical University Quanzhou Fujian China

**Keywords:** age‐period‐cohort, deaths, incidence, multiple myeloma, secular trends, United Nations Security Council

## Abstract

**Objectives:**

The objective of this study was to analyse trends in the burden of multiple myeloma (MM) in the five permanent members of the United Nations Security Council (China, the United States of America, the Russian Federation, England, and France) to understand country differences and factors.

**Methods:**

The Global Burden of Disease Study 2021 was utilised to analyse the incidence and mortality trends of MM from 1990 to 2021. An age‐period‐cohort model was employed to evaluate the disparities among nations, while an autoregressive integrated moving average (ARIMA) model was employed to forecast the impending developments over the subsequent 15 years.

**Results:**

The study found the burden of MM continues to increase in China and the Russian Federation, with significant increases particularly in older age groups. In the United States of America, age‐standardised incidence rates (ASIR) and age‐standardised death rates (ASDR) showed a downward trend, reflecting a reduction in the burden of disease. In England and France, ASIR increased overall, but ASDR remained stable. The burden of MM was found to be significantly correlated with age, period effect, and birth cohort through age‐period‐cohort analysis. The ARIMA model predictions indicated an increasing trend in ASIR and a stable ASDR in China and the Russian Federation, while in England, France, and the United States of America, there was an overall stabilisation of ASIR and ASDR.

**Conclusions:**

Significant differences in the burden of MM were found among the five countries. China and the Russian Federation are facing an increasing MM burden, particularly in older age groups, while the United States of America, England and France have made progress through early screening and treatment. The study suggests focusing on older populations, promoting early diagnosis and personalised treatment, and addressing lifestyle and environmental factors. Future research should examine risk factors across countries to inform public health policy.

## 1 Introduction

Multiple myeloma (MM) is a malignant tumour caused by an abnormal clonal proliferation of plasma cells in the bone marrow, which accounts for approximately 10% of all haematological malignancies and is the second most common haematological malignancy after malignant lymphoma [1, 2, 3]. Of all haematological malignancies, patients with MM have been reported to suffer the greatest burden of physical symptoms, including destructive bone lesions and pain, anaemia, hypercalcaemia, recurrent infections and renal impairment [4, 5]. In recent years, advanced therapeutic options, such as autologous stem cell transplantation, immunomodulatory drugs, targeted antibodies and proteasome inhibitors, and CAR‐T therapy, have significantly improved the 5‐year survival rate of MM, approaching 50%, with the introduction of autologous stem cell transplantation and the development of immunomodulatory drugs (e.g., lenalidomide, pomalidomide) and proteasome inhibitors being key breakthroughs [6, 7, 8].

Despite many efforts to improve the management of MM treatments, the number of MM cases and deaths remains high, as revealed by International Agency for Research on Cancer 2020 statistics [9]. There are significant regional variations in MM incidence and deaths globally, with the burden generally higher in developed countries than in developing countries [10]. For example, the age‐standardised incidence rates (ASIR) and age‐standardised death rates (ASDR) for MM in the United States of America in 2019 were 2.95 and 2.83/100,000, which were significantly higher than those in China (0.78 and 0.60/100,000, respectively), while in the Russian Federation the ASIR and ASDR were 1.80 and 1.30/100,000, respectively, and they were 4.34 and 2.89/100,000 in France and 5.22 and 3.04/100,000 in England, which highlight the significant differences in the burden of disease for MM between developed and developing countries [11]. With rapid economic and social development, the cancer spectrum in countries such as China and Russian Federation is gradually converging with that of developed countries such as the United States of America, England and France [9, 12]. In conclusion, MM is a lethal cancer with major health implications, not only as a leading cause of cancer‐related deaths but also as a major public health issue of growing concern in several countries worldwide.

China, the United States of America, the Russia Federation, England and France are located in Asia, North America and Europe, respectively, and as the world's major populous countries and economies, they are broadly represented in global public health affairs [13]. The five countries under scrutiny present a diverse array of demographics, healthcare systems, lifestyles and ethnic compositions, which may exert a profound influence on the epidemiological characteristics of MM [14]. Therefore, we hypothesise that there may be some heterogeneity among these five countries in terms of the disease burden of MM and its changing trends. It is important to acknowledge that these five countries are also permanent members of the United Nations Security Council, thereby possessing a significant degree of political influence in the realm of international health policy‐making.

Therefore, in this study, a comparative analysis was undertaken of the disease characteristics and trends associated with MM in these five countries. By using data based on the 2021 Global Burden of Disease Study (GBD 2021), we analysed the changes in MM incidence and deaths between 1990 and 2021 and used age‐period‐cohort modelling to reveal the differences in MM across countries in different age groups, periods and birth cohorts. In addition, we applied an autoregressive integrated moving average (ARIMA) model to predict trends in MM incidence and deaths in these five countries over the next 15 years. Understanding these factors is crucial for the diagnosis and treatment needs of MM and can reveal differences in MM incidence and deaths between these five countries, thus providing useful clues for further research into its underlying causes.

## 2 Methodology

### 2.1 Data Sources

We obtained data from GBD 2021, which assesses incidence, deaths, prevalence, years of life lost, years of life lived with disability, and disability‐adjusted life years associated with a wide range of diseases, stratified by age, sex, and region, integrating a wide range of inputs from surveys, censuses, vital statistics, and other sources of health data, to quantify the health burden of 371 diseases for 204 countries and territories burden [15]. The detailed methodology for the GBD study and cancer estimates has been covered in other topics [11]. We selected ‘China’, ‘the United States of America’, ‘England’, ‘France’ and ‘Russian Federation’ as locations, ‘MM’ as the cause of the disease, and incidence and death rates as measures. As incidence and death data in GBD 2021 were shown to be 0 until age 25 years, we age‐grouped from age 25 years, covering data from 15 age groups, each in 5‐year cohorts up to age 95+ years, and included data on MM patients of each sex. We used age‐standardised rates, incidence and deaths to analyse one aspect of the differences between the five countries. All rates were reported per 100,000 person‐years. Based on the inherent characteristics of the GBD database in terms of model selection, parameter estimation, and data quality and availability, 95% uncertainty intervals (UIs) were calculated from 1000 replications of the sample, with upper and lower bounds determined by the 2.5th and 97.5th percentiles of the uncertainty distribution, respectively [16]. Relevant data were anonymised and publicly available, and informed consent waivers were reviewed and approved by the University of Washington Institutional Review Board.

### 2.2 Statistical Analyses

All data analyses were performed using R 4.3.3 software. Furthermore, the Joinpoint 4.9 software was utilised to calculate the annual percentage change (APC) and the average annual percentage change (AAPC). The Intrinsic Estimation (IE) model was constructed utilising Stata MP 16 (64‐bit) software. Statistically speaking, a *p*‐value less than 0.05 was deemed to be statistically significant.

#### 2.2.1 Joinpoint Regression Analysis Model

The initial proposal of the joinpoint regression model was made by Kim in 1998 [17]. Temporal trends in incidence and deaths in five countries were explored using this model. The analysis identified significant turning points (joinpoints) in the change in incidence and death trends in each country and divided the overall trend into intervals defined by these joinpoints. The Joinpoint regression analyses in this study were modelled based on ASIR and mortality ASDR for each country stratified by sex provided by the GBD database, and the data used for the analyses were standardised rates integrated across all age groups. This allowed for a more detailed assessment of the epidemiological stages of the different phases of incidence and deaths, with the calculation of the APC, the AAPC, and their 95% confidence interval (CI) for each interval. In this regard, if the point estimate of APC/AAPC and the lower limit of its 95% CI are both greater than zero, it indicates a significant upward trend over the time period; conversely, if their upper limits are both less than zero, it indicates a downward trend [17]. The application of these techniques facilitates a more effective comparison of trends and the generation of more detailed results, particularly in the context of aggregation and comparison over specified time intervals.

#### 2.2.2 Age‐Period‐Cohort Analysis

An age‐period‐cohort analytical framework was used to profile the dataset, using age, period and birth cohort as the main independent variables, and to determine how they collectively affect incidence and deaths in MM [18]. In addition, to identify the independent effects of age, period, and birth cohort, we used an Intrinsic Estimation (IE) approach. The IE method is a specialised approach for distinguishing interrelated age, period, and cohort effects, which solves the problem of parameter unidentifiability by imposing specific constraints or assumptions in the age‐period‐cohort model to enable the estimation of the unique contribution of each factor and thus the identification of independent effects of age, period, and cohort [19]. Because uniformly formatted age and period data are critical to the structure of the age‐period‐cohort model, we stratified MM incidence, deaths, and demographic data according to predefined categories. First, we classified age into consecutive 5‐year intervals (e.g., 25–29 years, 30–34 years … 90–94 years and 95+ years). Second, we chose to use incidence and demographic data collected every 5 years between 1992 and 2021 to more accurately reflect changes across time. Finally, the birth cohort can be calculated by the formula “birth cohort = period − age”, covering different birth cohorts from 1897–1901 to 1992–1996. Estimated coefficients for age, period, and birth cohort effects are calculated by the IE method and converted to an exponential value [exp(coef) = e^coef^], which represents the relative risk (RR) of a particular age, period, or birth cohort relative to the overall average of all ages, periods, or birth cohorts [20]. The risk is thus relative to the overall average risk calculated for all possible age, period and birth cohort combinations within each country.

#### 2.2.3 Autoregressive Integrated Moving Average (ARIMA) Model

In recent years ARIMA models have been widely used in the previous literature to predict disease trends in populations, and well‐calibrated probabilistic predictions have been obtained [21, 22, 23]. In this study, we employed ARIMA models for ASIR and ASDR for five countries for the period 1990 to 2021, based on the projected data for these countries from 2022 to 2036. The models were implemented by using a statistical package to carry out data fitting and forecasting.

## 3 Results

### 3.1 Descriptive Analysis

**FIGURE 1:**
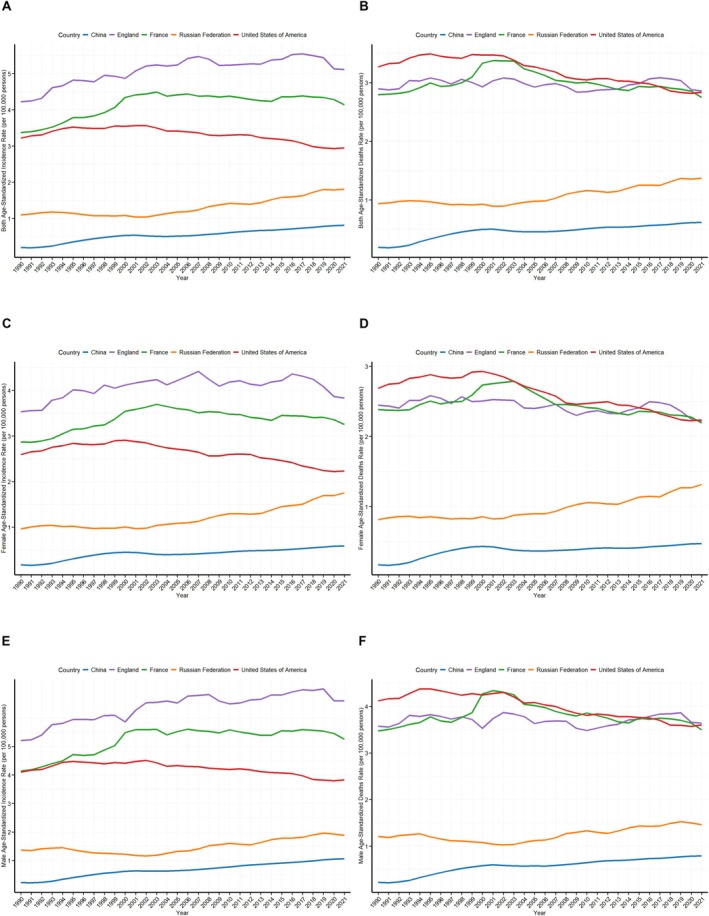
The trends in country‐specific age‐standardised incidence and age‐standardised death rates of multiple myeloma from 1990 to 2021 for both sexes (A, B), females (C, D) and males (E, F).

Figure 1 and Tables 1 and 2 show the incidence and death rates of MM in the five countries (China, Russian Federation, France, the United States of America and England) analysed for the period 1990 versus 2021, revealing the epidemiological trends in these countries between different sexes and age groups. The *p*‐values in Table 1 were used to assess the statistical significance of the overall trend in ASIR and ASDR between 1990 and 2021.

**TABLE 1:** Age‐standardised incidence rates (ASIR) and age‐standardised death rates (ASDR) for multiple myeloma in 1990 and 2021 and their annual percentage changes from 1990 to 2021 in five countries.

	China				Russian Federation				France				United States of America				England			
	Rates in 1990, 95% UI	Rates in 2021, 95% UI	*p*	AAPC_95CI	Rates in 1990, 95% UI	Rates in 2021, 95% UI	*p*	AAPC_95CI	Rates in 1990, 95% UI	Rates in 2021, 95% UI	*p*	AAPC_95CI	Rates in 1990, 95% UI	Rates in 2021, 95% UI	*p*	AAPC_95CI	Rates in 1990, 95% UI	Rates in 2021, 95% UI	*p*	AAPC_95CI
ASIR																				
Both	0.2 (0.13, 0.39)	0.81 (0.52, 1.07)	0	4.74 (4.18, 5.29)	1.1 (1.07, 1.13)	1.81 (1.66, 1.96)	0	1.74 (1.20, 2.29)	3.37 (3.11, 3.62)	4.14 (3.57, 4.81)	0	0.78 (0.48, 1.09)	3.22 (3.05, 3.32)	2.95 (2.7, 3.09)	0.003471	−0.33 (−0.55, −0.11)	4.22 (4.04, 4.36)	5.11 (4.74, 5.36)	0.033204	0.64 (0.05, 1.23)
Female	0.17 (0.1, 0.42)	0.59 (0.25, 0.84)	0	4.09 (3.08, 5.11)	0.96 (0.93, 0.99)	1.75 (1.56, 1.94)	0	2.03 (1.34, 2.73)	2.87 (2.55, 3.19)	3.25 (2.61, 3.95)	4.9e‐05	0.47 (0.24, 0.70)	2.59 (2.41, 2.7)	2.23 (2, 2.37)	3e‐06	−0.55 (−0.78, −0.32)	3.53 (3.33, 3.7)	3.83 (3.42, 4.09)	0.254952	0.29 (−0.21, 0.78)
Male	0.23 (0.15, 0.52)	1.06 (0.6, 1.48)	0	5.18 (4.79, 5.58)	1.37 (1.31, 1.42)	1.89 (1.64, 2.08)	0	1.28 (0.79, 1.77)	4.14 (3.75, 4.52)	5.25 (4.27, 6.31)	0	0.89 (0.55, 1.23)	4.1 (3.94, 4.21)	3.83 (3.56, 4)	0.000885	−0.26 (−0.41, −0.11)	5.21 (5.02, 5.38)	6.6 (6.16, 6.93)	0.014161	0.80 (0.16, 1.45)
ASDR																				
Both	0.19 (0.13, 0.39)	0.62 (0.4, 0.81)	0	3.91 (2.79, 5.05)	0.94 (0.91, 0.96)	1.37 (1.26, 1.48)	5e‐06	1.33 (0.76, 1.90)	2.8 (2.59, 2.95)	2.75 (2.35, 3.11)	0.770813	0.05 (−0.28, 0.37)	3.27 (3.07, 3.38)	2.84 (2.58, 2.99)	0.000752	−0.49 (−0.77, −0.21)	2.89 (2.77, 2.96)	2.86 (2.61, 2.99)	0.901553	−0.03 (−0.50, 0.44)
Female	0.17 (0.1, 0.42)	0.47 (0.2, 0.67)	0	3.36 (2.36, 4.37)	0.81 (0.79, 0.84)	1.31 (1.17, 1.44)	2.4e‐05	1.57 (0.84, 2.31)	2.38 (2.17, 2.58)	2.19 (1.79, 2.64)	0.224935	−0.16 (−0.42, 0.10)	2.69 (2.48, 2.81)	2.24 (1.98, 2.38)	9.4e‐05	−0.64 (−0.96, −0.32)	2.45 (2.3, 2.52)	2.22 (1.96, 2.35)	0.284591	−0.29 (−0.81, 0.24)
Male	0.22 (0.14, 0.52)	0.79 (0.46, 1.11)	0	4.25 (3.77, 4.74)	1.21 (1.16, 1.25)	1.46 (1.28, 1.62)	1.6e‐05	0.81 (0.44, 1.18)	3.48 (3.19, 3.7)	3.5 (2.94, 4.06)	0.606783	0.10 (−0.28, 0.49)	4.12 (3.94, 4.24)	3.6 (3.34, 3.77)	0	−0.43 (−0.58, −0.28)	3.58 (3.47, 3.65)	3.64 (3.39, 3.79)	0.797122	0.07 (−0.49, 0.64)

**TABLE 2:** Multiple myeloma incidence and death rates for different age groups in the five countries in 2021 and their percentage change 1990–2021.

Categories	China		United States of America		Russian Federation		England		France	
	Rates in 2021, 95% UI	AAPC_95CI	Rates in 2021, 95% UI	AAPC_95CI	Rates in 2021, 95% UI	AAPC_95CI	Rates in 2021, 95% UI	AAPC_95CI	Rates in 2021, 95% UI	AAPC_95CI
Incidence										
25–29 years	0.15 (0.07, 0.21)	6.04 (4.97, 7.11)	0.03 (0.03, 0.04)	0.98 (−0.04, 2.01)	0.05 (0.05, 0.06)	0.59 (0.17, 1.01)	0.06 (0.06, 0.07)	1.52 (0.51, 2.54)	0.07 (0.05, 0.1)	0.40 (0.01, 0.79)
30–34 years	0.17 (0.09, 0.24)	5.45 (4.38, 6.52)	0.1 (0.09, 0.1)	1.12 (0.11, 2.14)	0.12 (0.11, 0.13)	−0.23 (−1.24, 0.80)	0.2 (0.17, 0.23)	1.47 (0.66, 2.28)	0.21 (0.14, 0.29)	0.83 (0.48, 1.18)
35–39 years	0.3 (0.17, 0.4)	5.20 (4.29, 6.12)	0.26 (0.24, 0.27)	1.09 (0.59, 1.59)	0.32 (0.29, 0.34)	−0.28 (−0.71, 0.15)	0.56 (0.49, 0.64)	1.42 (0.96, 1.89)	0.47 (0.32, 0.66)	0.41 (−0.25, 1.06)
40–44 years	0.47 (0.27, 0.65)	5.14 (3.84, 6.45)	0.61 (0.57, 0.65)	0.56 (−0.11, 1.23)	0.74 (0.67, 0.82)	−0.84 (−1.23, −0.44)	1.29 (1.13, 1.47)	1.36 (0.86, 1.86)	1.07 (0.76, 1.52)	0.29 (−0.16, 0.75)
45–49 years	0.67 (0.39, 0.91)	4.81 (3.97, 5.65)	1.38 (1.31, 1.46)	0.57 (0.07, 1.07)	1.5 (1.36, 1.66)	−1.03 (−1.30, −0.76)	3.06 (2.7, 3.45)	1.19 (0.85, 1.53)	2.31 (1.62, 3.18)	0.05 (−0.51, 0.61)
50–54 years	1.27 (0.76, 1.72)	5.04 (3.77, 6.32)	2.97 (2.83, 3.13)	0.95 (0.04, 1.86)	3.16 (2.87, 3.51)	−1.07 (−1.29, −0.85)	6.14 (5.46, 6.86)	0.56 (−0.19, 1.32)	4.62 (3.31, 6.44)	0.39 (−0.04, 0.82)
55–59 years	1.91 (1.18, 2.6)	4.34 (2.89, 5.82)	5.18 (4.98, 5.4)	1.16 (0.83, 1.50)	5.32 (4.76, 5.83)	−1.10 (−1.20, −0.99)	8.93 (8.21, 9.64)	−0.21 (−0.44, 0.02)	7.4 (5.26, 10.2)	0.51 (0.23, 0.80)
60–64 years	2.98 (1.8, 4.01)	4.68 (3.54, 5.82)	8.79 (8.4, 9.18)	1.47 (0.31, 2.64)	7.95 (7.22, 8.77)	−1.00 (−1.16, −0.84)	15.28 (14.11, 16.46)	−0.13 (−0.76, 0.51)	12.55 (9.04, 16.79)	0.45 (−0.10, 1.00)
65–69 years	3.99 (2.42, 5.33)	4.60 (3.38, 5.84)	13.31 (12.63, 13.9)	1.87 (1.22, 2.52)	10.78 (9.88, 11.81)	−0.82 (−1.12, −0.51)	21.71 (20.07, 23.21)	−0.08 (−0.67, 0.51)	19.3 (14.4, 24.95)	0.61 (0.19, 1.04)
70–74 years	5.42 (3.57, 7.17)	4.72 (3.83, 5.63)	20.06 (18.66, 21.02)	2.16 (1.63, 2.69)	12.6 (11.47, 13.79)	−0.41 (−0.79, −0.02)	33.1 (30.68, 35.26)	0.56 (0.26, 0.87)	28.63 (21.77, 37.05)	0.83 (0.36, 1.31)
75–79 years	5.63 (3.84, 7.43)	4.70 (3.91, 5.51)	28.6 (25.79, 30.12)	3.07 (1.87, 4.30)	12.64 (11.6, 13.68)	0.04 (−0.28, 0.36)	48.39 (43.71, 51.89)	0.95 (0.55, 1.36)	38.44 (29.2, 49.46)	1.16 (1.00, 1.32)
80–84 years	5.06 (3.12, 6.62)	5.15 (3.79, 6.52)	36.81 (30.83, 39.89)	2.98 (1.19, 4.80)	9.93 (8.92, 10.75)	0.51 (0.29, 0.73)	63.34 (54.32, 69.24)	1.42 (1.00, 1.85)	44.4 (32.78, 58.67)	0.86 (0.27, 1.45)
85–89 years	5.91 (3.64, 7.57)	5.50 (4.45, 6.55)	43.93 (35.08, 48.65)	2.69 (1.37, 4.03)	5.19 (4.38, 5.81)	1.05 (0.73, 1.37)	77.36 (62.79, 85.63)	1.85 (0.81, 2.90)	55.27 (39.19, 73.96)	0.54 (−0.46, 1.56)
90–94 years	4.59 (2.8, 6.17)	4.96 (3.87, 6.06)	43.29 (32.9, 49.07)	1.87 (0.90, 2.85)	4.21 (3.47, 4.74)	1.40 (0.90, 1.90)	72.65 (57.9, 81.04)	1.95 (1.47, 2.42)	71.2 (51.55, 93.03)	1.39 (0.73, 2.06)
95+ years	2.39 (1.31, 3.31)	4.62 (3.53, 5.72)	32 (22.73, 36.84)	1.30 (−0.55, 3.20)	2.52 (1.86, 2.9)	1.46 (0.36, 2.57)	53.41 (41.79, 59.52)	2.31 (1.74, 2.89)	52.01 (37.8, 66.12)	1.79 (−0.11, 3.72)
Deaths										
25–29 years	0.09 (0.04, 0.12)	4.89 (4.07, 5.71)	0.02 (0.02, 0.03)	0.11 (−0.17, 0.39)	0.03 (0.03, 0.03)	0.32 (−0.63, 1.28)	0.02 (0.02, 0.02)	−0.01 (−0.98, 0.96)	0.03 (0.02, 0.03)	−0.92 (−1.34, −0.50)
30–34 years	0.1 (0.05, 0.14)	4.30 (3.18, 5.42)	0.07 (0.07, 0.07)	−0.67 (−1.56, 0.22)	0.07 (0.06, 0.08)	0.47 (−0.54, 1.50)	0.06 (0.06, 0.06)	0.06 (−0.61, 0.74)	0.08 (0.06, 0.1)	−0.50 (−0.71, −0.28)
35–39 years	0.16 (0.1, 0.22)	4.04 (2.85, 5.25)	0.17 (0.16, 0.18)	−0.93 (−1.64, −0.21)	0.18 (0.16, 0.19)	0.37 (−0.22, 0.96)	0.16 (0.16, 0.17)	0.15 (−0.73, 1.03)	0.16 (0.13, 0.2)	−1.00 (−1.55, −0.45)
40–44 years	0.28 (0.16, 0.37)	3.98 (2.65, 5.31)	0.42 (0.4, 0.44)	−1.16 (−2.02, −0.29)	0.43 (0.4, 0.47)	0.25 (−0.60, 1.12)	0.4 (0.39, 0.41)	−0.02 (−0.95, 0.92)	0.39 (0.32, 0.47)	−1.00 (−1.39, −0.61)
45–49 years	0.4 (0.24, 0.54)	3.72 (2.87, 4.57)	0.97 (0.92, 1.01)	−1.54 (−2.02, −1.06)	0.9 (0.82, 0.98)	−0.14 (−0.68, 0.40)	0.97 (0.94, 0.99)	−0.32 (−0.84, 0.21)	0.86 (0.72, 1.01)	−1.14 (−1.74, −0.55)
50–54 years	0.77 (0.46, 1.05)	3.56 (2.31, 4.82)	2.1 (2.03, 2.19)	−1.62 (−2.04, −1.21)	1.92 (1.74, 2.09)	0.37 (−0.42, 1.16)	2 (1.94, 2.05)	−0.74 (−1.51, 0.04)	1.75 (1.46, 2.07)	−0.92 (−1.60, −0.25)
55–59 years	1.24 (0.78, 1.71)	3.70 (2.58, 4.83)	3.93 (3.79, 4.06)	−1.57 (−1.70, −1.44)	3.42 (3.1, 3.71)	0.56 (0.28, 0.84)	3.45 (3.34, 3.56)	−1.29 (−1.41, −1.18)	3.18 (2.7, 3.75)	−0.87 (−1.04, −0.69)
60–64 years	2.04 (1.23, 2.74)	3.73 (2.60, 4.87)	7.11 (6.84, 7.36)	−1.38 (−1.71, −1.05)	5.49 (4.99, 6)	0.78 (−0.27, 1.84)	6.41 (6.19, 6.6)	−1.08 (−1.36, −0.80)	6.15 (5.08, 7.19)	−0.57 (−1.07, −0.07)
65–69 years	2.96 (1.81, 3.97)	3.73 (2.48, 5.00)	11.5 (10.93, 11.94)	−1.14 (−1.35, −0.94)	8.14 (7.49, 8.87)	1.26 (0.65, 1.87)	10.52 (10.09, 10.88)	−1.00 (−1.65, −0.35)	10.83 (9.13, 12.64)	−0.20 (−0.71, 0.32)
70–74 years	4.36 (2.86, 5.8)	3.95 (3.10, 4.81)	18.83 (17.49, 19.66)	−0.70 (−0.98, −0.42)	10.49 (9.57, 11.46)	1.69 (1.22, 2.17)	18.07 (17.06, 18.73)	−0.23 (−0.48, 0.02)	18.44 (15.29, 21.65)	0.04 (−0.44, 0.52)
75–79 years	5.05 (3.4, 6.65)	4.02 (3.33, 4.71)	29.25 (26.57, 30.67)	−0.19 (−0.47, 0.09)	11.54 (10.6, 12.52)	2.68 (1.53, 3.84)	29.37 (26.99, 30.65)	0.08 (−0.48, 0.65)	28.33 (23.14, 33.72)	0.43 (−0.09, 0.96)
80–84 years	5.1 (3.17, 6.63)	4.54 (3.17, 5.94)	41.66 (35.29, 45.01)	0.32 (0.10, 0.53)	10.17 (9.15, 11.01)	2.68 (1.09, 4.30)	44.91 (39.02, 47.92)	0.83 (0.34, 1.32)	38.8 (29.3, 48.06)	0.31 (−0.25, 0.88)
85–89 years	6.45 (3.92, 8.36)	4.93 (3.88, 5.99)	53.91 (43.16, 59.6)	0.88 (0.59, 1.17)	5.81 (5.01, 6.47)	2.43 (1.17, 3.70)	62.03 (51.52, 67)	1.46 (0.39, 2.55)	54.55 (39.15, 71.05)	0.07 (−0.75, 0.90)
90–94 years	5.51 (3.38, 7.35)	4.06 (1.70, 6.48)	58.33 (44.3, 65.87)	1.24 (0.71, 1.76)	5.12 (4.22, 5.76)	1.57 (0.64, 2.51)	63.76 (51.59, 70.08)	1.45 (0.75, 2.14)	78.67 (60.63, 98.88)	0.92 (0.25, 1.59)
95+ years	3.75 (2.02, 5.22)	4.54 (3.44, 5.65)	50.86 (36.07, 58.45)	1.44 (0.34, 2.54)	3.95 (2.92, 4.54)	1.26 (−0.61, 3.17)	77.58 (60.35, 86.17)	2.18 (1.61, 2.75)	79.74 (59.59, 102.38)	1.70 (−0.17, 3.61)

In China, the ASIR of MM increased from 0.2 (95% UI: 0.13, 0.39) in 1990 to 0.81 (95% UI: 0.52, 1.07) in 2021, showing a gradual upward trend over the past three decades (Table 1). In contrast, the ASDR increased from 0.19 (95% UI: 0.13, 0.39) in 1990 to 0.62 (95% UI: 0.4, 0.81) in 2021, reflecting a continuous increase in deaths (Table 1). The 85–89 years group showed the highest incidence rate (5.91, 95% UI: 3.64, 7.57) and death rate (6.45, 95% UI: 3.92, 8.36) among all groups (Table 2). Similar trends were observed in other countries, although the specific situation varied from country to country.

In the United States of America, both ASIR and ASDR for MM have declined, from 3.22 (95% UI: 3.05, 3.32) in 1990 to 2.95 (95% UI: 2.7, 3.09) in 2021, and the death rate has decreased from 3.27 (95% UI: 3.07, 3.38) to 2.84 (95% UI: 2.58, 2.99) (Table 1). Despite the overall decreasing trend, the highest incidence was still found in the 85–89 years group (43.93, 95% UI: 35.08, 48.65) and the highest deaths in the 90–94 years group (58.33, 95% UI: 44.3, 65.87) (Table 2).

In England and France, the incidence of MM has shown an increasing trend, with ASIR increasing from 4.22 (95% UI: 4.04, 4.36) in 1990 to 5.11 (95% UI: 4.74, 5.36) and from 3.37 (95% UI: 3.11, 3.62) to 4.14 (95% UI: 3.57, 4.81) in 2021, while deaths remained relatively stable (Table 1). In England, the highest prevalence was found in the 85–89 age group (77.36, 95% UI: 62.79, 85.63), while in France, the highest deaths were found in the 95+ age group (79.74, 95% UI: 59.59, 102.38) (Table 2).

In the Russian Federation, ASIR increased from 1.1 (95% UI: 1.07, 1.13) in 1990 to 1.81 (95% UI: 1.66, 1.96) in 2021, especially in the 75–79 years group, where incidence (12.64, 95% UI: 11.6, 13.68) and deaths (11.54, 95% UI. 10.6, 12.52) were more prominent (Tables 1 and 2).

### 3.2 Joinpoint Regression Analysis

**FIGURE 2:**
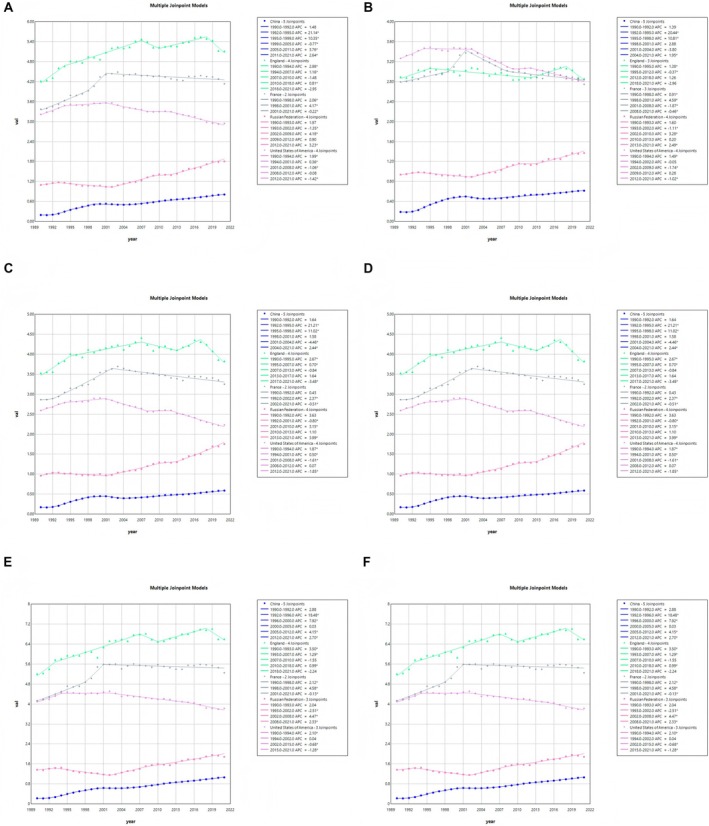
Joinpoint regression analysis of age‐standardised incidence rates (both sex (A), female (C) and male (E)), and age‐standardised death rates (both sex (B), female (D) and male (F)) for multiple myeloma in the five countries from 1990 to 2021. *With significance, *p* < 0.05.

The AAPC in ASIR and ASDR for the five countries is presented in Figure 2 and in Table 1.

**TABLE 3:** Percentage change in multiple myeloma incidence and death rates in five countries by Period Group, 1990–2021.

Sex	Incidence									
	China		France		England		United States of America		Russian Federation	
	Trend/Period	APC (95% CI)	Trend/Period	APC (95% CI)	Trend/Period	APC (95% CI)	Trend/Period	APC (95% CI)	Trend/Period	APC (95% CI)
Both	1990–1992	1.48 (−4.30–7.61)	1990–1998	2.06 (1.75–2.38)	1990–1994	2.98 (1.72–4.25)	1990–1994	1.99 (1.34–2.64)	1990–1993	1.97 (−0.50–4.50)
	1992–1995	21.14 (16.55–25.92)	1998–2001	4.17 (1.02–7.42)	1994–2007	1.16 (0.91–1.41)	1994–2001	0.36 (0.00–0.73)	1993–2002	−1.35 (−1.81 to −0.89)
	1995–1999	10.35 (9.00–11.73)	2001–2021	−0.22 (−0.33 to −0.11)	2007–2010	−1.48 (−6.28–3.56)	2001–2008	−1.06 (−1.45 to −0.66)	2002–2009	4.18 (3.42–4.95)
	1999–2005	−0.77 (−1.32 to −0.22)			2010–2018	0.81 (0.06–1.57)	2008–2012	−0.08 (−1.37–1.23)	2009–2012	0.90 (−3.62–5.63)
	2005–2011	3.76 (3.26–4.26)			2018–2021	−2.95 (−5.90–0.09)	2012–2021	−1.42 (−1.69 to −1.15)	2012–2021	3.23 (2.61–3.85)
	2011–2021	2.64 (2.44–2.85)								
Female	1990–1992	1.64 (−7.47–11.63)	1990–1992	0.43 (−2.62–3.57)	1990–1995	2.67 (1.76–3.58)	1990–1994	1.87 (1.18–2.56)	1990–1992	3.63 (−1.73–9.29)
	1992–1995	21.21 (13.74–29.16)	1992–2002	2.37 (2.05–2.69)	1995–2007	0.70 (0.41–1.00)	1994–2001	0.50 (0.13–0.87)	1992–2001	−0.80 (−1.35 to −0.25)
	1995–1998	11.02 (7.08–15.10)	2002–2021	−0.51 (−0.65 to −0.38)	2007–2013	−0.84 (−1.94–0.28)	2001–2008	−1.61 (−2.01 to −1.21)	2001–2010	3.15 (2.59–3.71)
	1998–2001	1.58 (−2.28–5.59)			2013–2017	1.64 (−1.20–4.56)	2008–2012	0.07 (−1.31–1.45)	2010–2013	1.10 (−4.52–7.04)
	2001–2004	−4.46 (−7.69 to −1.13)			2017–2021	−3.48 (−5.33 to −1.58)	2012–2021	−1.85 (−2.12 to −1.57)	2013–2021	3.99 (3.06–4.93)
	2004–2021	2.44 (2.29–2.59)								
Male	1990–1992	2.88 (−1.80–7.79)	1990–1998	2.12 (1.75–2.49)	1990–1993	3.50 (1.19–5.85)	1990–1994	2.10 (1.48–2.72)	1990–1993	2.04 (−1.74–5.96)
	1992–1996	18.48 (16.62–20.37)	1998–2001	4.58 (1.04–8.24)	1993–2007	1.29 (1.03–1.56)	1994–2002	0.04 (−0.24–0.32)	1993–2002	−2.51 (−3.15 to −1.87)
	1996–2000	7.92 (6.73–9.12)	2001–2021	−0.13 (−0.26 to −0.01)	2007–2010	−1.54 (−6.43–3.60)	2002–2015	−0.68 (−0.83 to −0.53)	2002–2008	4.47 (3.07–5.89)
	2000–2005	0.03 (−0.55–0.61)			2010–2018	0.99 (0.15–1.84)	2015–2021	−1.28 (−1.81 to −0.75)	2008–2021	2.33 (1.94–2.72)
	2005–2012	4.15 (3.84–4.46)			2018–2021	−2.24 (−5.63–1.27)				
	2012–2021	2.70 (2.51–2.90)								

Sex	Deaths									
	China		France		England		United States of America		Russian Federation	
	Trend/Period	APC (95% CI)	Trend/Period	APC (95% CI)	Trend/Period	APC (95% CI)	Trend/Period	APC (95% CI)	Trend/Period	APC (95% CI)
Both	1990–1992	1.39 (−8.39–12.22)	1990–1998	0.91 (0.59–1.23)	1990–1995	1.38 (0.48–2.29)	1990–1994	1.49 (0.84–2.14)	1990–1993	1.60 (−0.71–3.97)
	1992–1995	20.44 (12.56–28.88)	1998–2001	4.59 (1.53–7.75)	1995–2012	−0.37 (−0.57 to −0.17)	1994–2002	−0.05 (−0.33–0.23)	1993–2002	−1.11 (−1.56 to −0.66)
	1995–1998	10.81 (6.43–15.38)	2001–2008	−1.87 (−2.44 to −1.29)	2012–2018	1.26 (−0.28–2.83)	2002–2009	−1.74 (−2.14 to −1.34)	2002–2010	3.28 (2.68–3.88)
	1998–2001	2.88 (−1.96–7.95)	2008–2021	−0.46 (−0.71 to −0.21)	2018–2021	−2.96 (−6.53–0.74)	2009–2012	0.28 (−2.37–3.01)	2010–2013	0.20 (−4.77–5.43)
	2001–2004	−3.80 (−7.74–0.31)					2012–2021	−1.02 (−1.29 to −0.76)	2013–2021	2.49 (1.76–3.23)
	2004–2021	1.95 (1.80–2.10)								
Female	1990–1992	1.17 (−9.12–12.61)	1990–1998	0.81 (0.51–1.12)	1990–1995	1.26 (0.36–2.17)	1990–1994	1.60 (1.03–2.17)	1990–1992	2.43 (−2.48–7.59)
	1992–1995	21.59 (13.05–30.77)	1998–2002	3.06 (1.59–4.54)	1995–2013	−0.54 (−0.71 to −0.36)	1994–1997	−0.42 (−2.13–1.33)	1992–2001	−0.36 (−0.83–0.12)
	1995–1999	9.38 (7.13–11.69)	2002–2007	−2.79 (−3.78 to −1.79)	2013–2017	1.95 (−1.40–5.41)	1997–2000	1.38 (−0.43–3.23)	2001–2006	1.88 (0.53–3.24)
	1999–2005	−3.25 (−4.11 to −2.38)	2007–2021	−0.67 (−0.89 to −0.45)	2017–2021	−3.23 (−5.48 to −0.92)	2000–2009	−2.00 (−2.21 to −1.78)	2006–2010	3.84 (1.33–6.40)
	2005–2021	1.59 (1.41–1.78)					2009–2012	0.50 (−1.80–2.85)	2010–2013	−0.42 (−6.00–5.48)
							2012–2021	−1.37 (−1.60 to −1.15)	2013–2021	3.01 (2.15–3.89)
	1990–1992	2.55 (−3.16–8.60)	1990–1998	0.88 (0.49–1.28)	1990–1995	1.63 (0.93–2.33)	1990–1994	1.49 (0.85–2.13)	1990–1994	0.77 (−1.03–2.60)
	1992–1996	18.04 (15.72–20.40)	1998–2001	5.32 (1.73–9.03)	1995–2000	−1.13 (−2.24 to −0.01)	1994–2002	−0.30 (−0.59 to −0.01)	1994–2002	−2.50 (−3.21 to −1.79)
	1996–2000	7.37 (5.91–8.85)	2001–2007	−2.09 (−2.98 to −1.20)	2000–2003	1.93 (−2.22–6.25)	2002–2009	−1.29 (−1.70 to −0.87)	2002–2009	3.41 (2.50–4.32)
	2000–2006	−0.64 (−1.15 to −0.13)	2007–2021	−0.48 (−0.74 to −0.23)	2003–2010	−1.35 (−2.09 to −0.59)	2009–2021	−0.64 (−0.82 to −0.47)	2009–2021	1.57 (1.18–1.97)
	2006–2012	3.11 (2.62–3.61)			2010–2018	1.29 (0.53–2.07)				
	2012–2021	1.65 (1.41–1.88)			2018–2021	−2.18 (−5.28–1.02)				

China's ASIR and ASDR for MM showed a significant growth trend and the fastest growth rate among the five countries. The average annual percentage change (AAPC) for ASIR reached 4.74 (95% CI: 4.18, 5.29), and the AAPC for ASDR was 3.91 (95% CI: 2.79, 5.05) (Table 1). The growth rate of ASIR was particularly significant between 1992 and 1995, with an APC of 21.14 (95% CI: 16.55, 25.92) (Table 3), and ASIR continued to grow rapidly between 2011 and 2021 (Figure 2). In addition, the increase in incidence was particularly marked in younger patients, with an AAPC of 6.04 (95% CI: 4.97, 7.11; Table 2) in the 25–29 age group.

In contrast to China, both ASIR and ASDR for both sexes in the United States of America showed a declining trend, with an AAPC of −0.33 (95% CI: −0.55, −0.11) for ASIR and an AAPC of −0.49 (95% CI: −0.77, −0.21) for ASDR (Table 1). ASIR declined most significantly in the United States of America between 2012 and 2021, with an APC of −1.42 (95% CI: −1.69, −1.15), while ASDR declined significantly between 2002 and 2009, with an APC of −1.74 (95% CI: −2.14, −1.34) (Table 3), showing a trend towards reducing the burden of disease (Figure 2).

The ASIR and ASDR for both sexes in the Russian Federation, while also trending upwards, are relatively small and growing steadily, with an AAPC of 1.74 (95% CI: 1.20, 2.29) for the ASIR and 1.33 (95% CI: 0.76, 1.90) for the ASDR (Table 1). Its growth was mainly concentrated between 1993 and 2020, with an overall smooth curve and small fluctuations (Figure 2).

In contrast, trends in ASIR and ASDR were generally stable for both sexes in England and France, although there were some differences between the two countries. The AAPC for ASIR in England was 0.64 (95% CI: 0.05, 1.23), and the AAPC for ASDR was −0.03 (95% CI: −0.50, 0.44) (Table 1). Notably, the ASIR in England showed a significant decline between 2018 and 2021. In contrast, the AAPC of 0.78 (95% CI: 0.48, 1.09) for ASIR and 0.05 (95% CI: −0.28, 0.37) for ASDR in France (Table 1) showed the smallest change, with the curves exhibiting a more stable trend (Figure 2).

Changes in incidence and deaths are generally more pronounced in males than in females, with the fastest increases in Chinese males, with significant increases in ASIR and ASDR (Figure 2); the most pronounced decreases have been in males in the United States of America, where ASIR and ASDR have continued to decline over the last decade (Figure 2); the Russian Federation and England, where males have slightly higher incidence than females, have seen smaller changes (Figure 2); and in France, where incidence and deaths for both sexes have changed in morbidity, with either a more moderate increase or decline for females than for males (Figure 2). Overall, the change in trend is more pronounced for men, while the curve for women is relatively smooth. But the burden is still greater for men than for women (Figure 2).

### 3.3 Age‐Period‐Cohort Analysis With the Intrinsic Estimator

**FIGURE 3:**
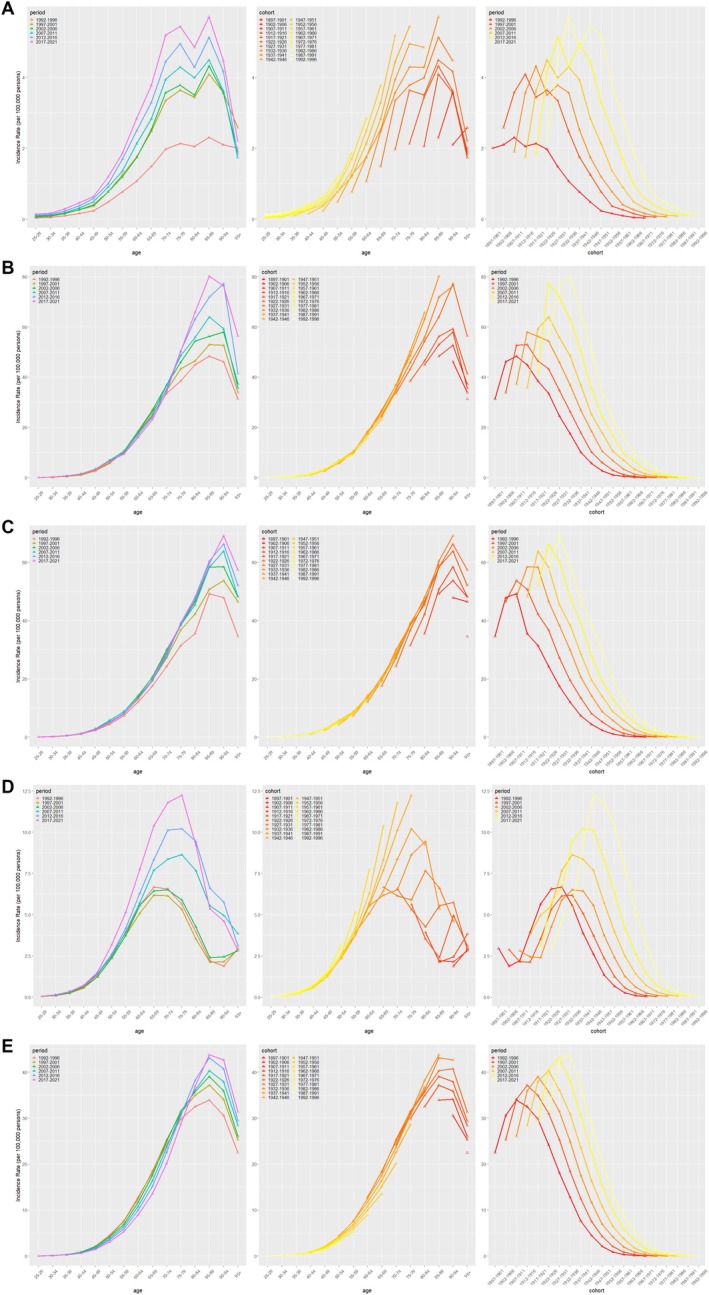
Age‐period‐specific incidence rates of multiple myeloma in the total populations of China (A), England (B), France (C), Russian Federation (D) and the United States of America (E).

**FIGURE 4:**
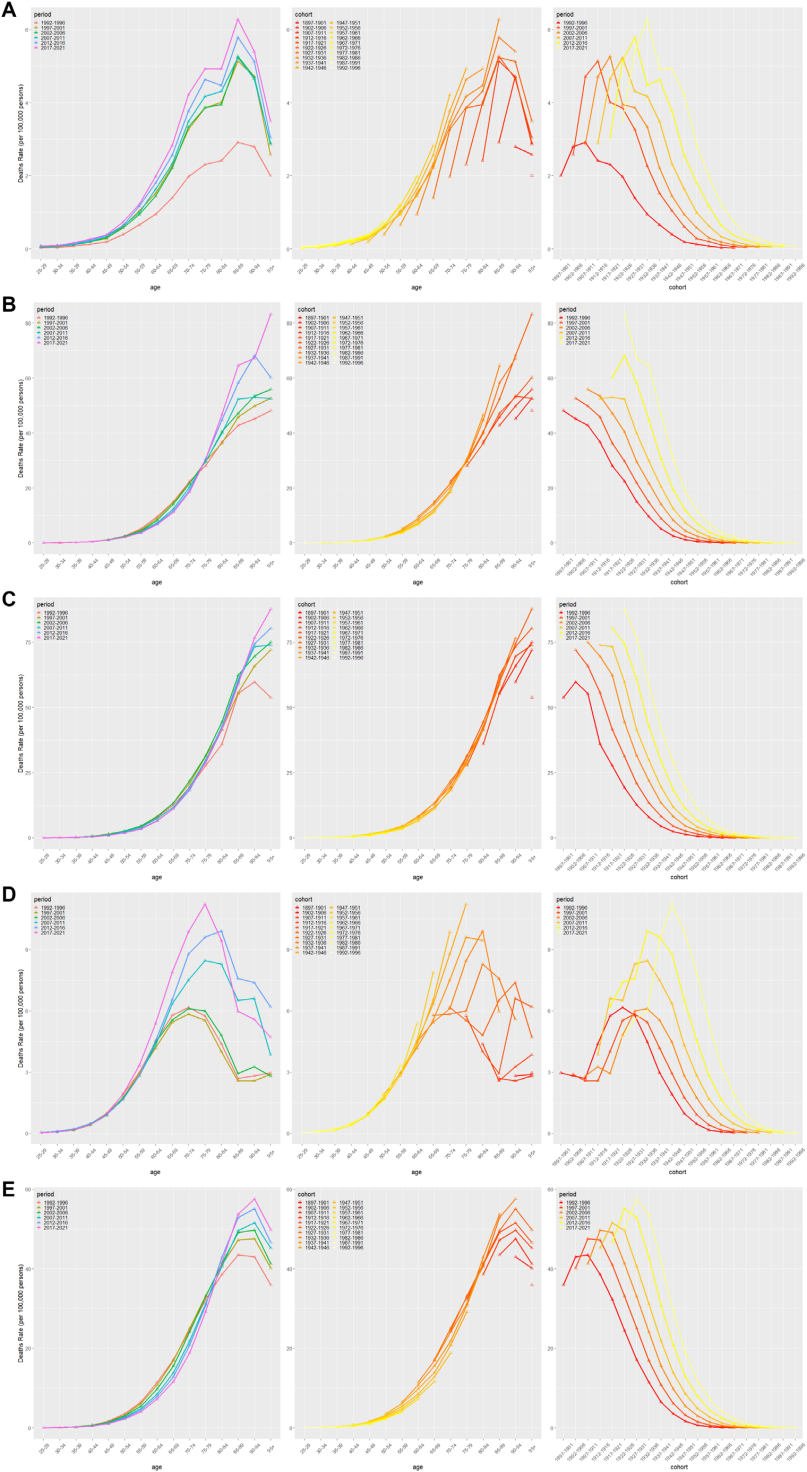
Age‐period‐specific death rates for multiple myeloma in the total populations of China (A), England (B), France (C), Russian Federation (D) and the United States of America (E).

**FIGURE 5:**
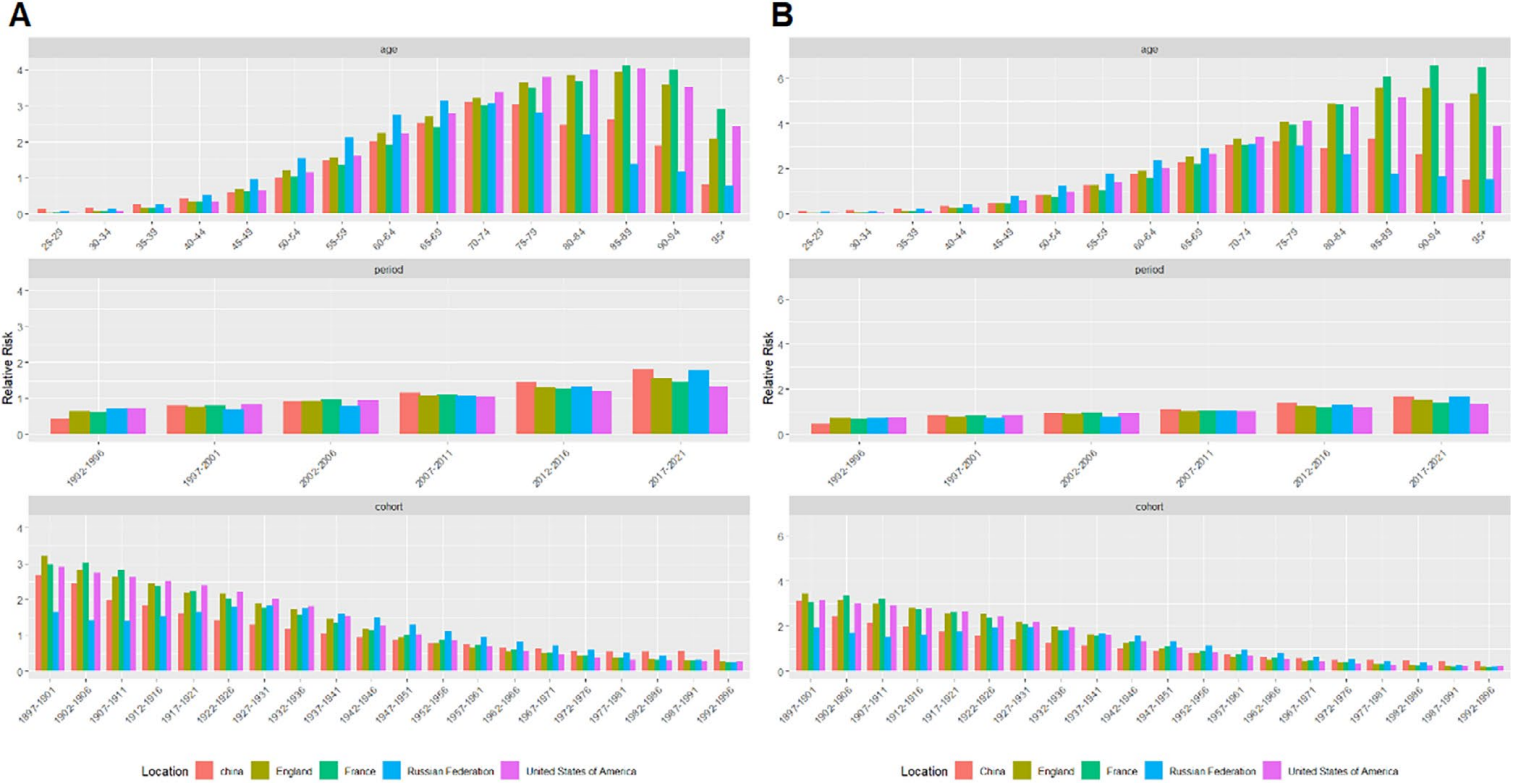
Relative risk of incidence (A) and deaths (B) of multiple myeloma due to age, period and cohort effects in the permanent members of the five countries from 1992 to 2021.

The two‐by‐two relationships for age, period, and cohort are shown in Figure 3 and Figure 4. The RRs of MM due to age, period, and cohort effects for the five countries are presented in Figure 5 and Table 4.

**TABLE 4:** Relative risk of multiple myeloma due to age, period and cohort effects, five countries, 1992–2021.

Categories	Incidence									
	China		France		England		United States of America		Russian Federation	
	*p*	RR (95% CI)	*p*	RR (95% CI)	*p*	RR (95% CI)	*p*	RR (95% CI)	*p*	RR (95% CI)
Age effects										
25–29	< 0.001	0.13 (0.11, 0.15)	< 0.001	0.03 (0.02, 0.04)	< 0.001	0.02 (0.02, 0.04)	< 0.001	0.02 (0.02, 0.03)	< 0.001	0.08 (0.06, 0.1)
30–34	< 0.001	0.15 (0.13, 0.18)	< 0.001	0.07 (0.06, 0.09)	< 0.001	0.07 (0.06, 0.08)	< 0.001	0.06 (0.06, 0.07)	< 0.001	0.13 (0.11, 0.16)
35–39	< 0.001	0.27 (0.24, 0.31)	< 0.001	0.17 (0.15, 0.19)	< 0.001	0.16 (0.14, 0.19)	< 0.001	0.15 (0.14, 0.17)	< 0.001	0.28 (0.24, 0.32)
40–44	< 0.001	0.43 (0.39, 0.47)	< 0.001	0.33 (0.3, 0.37)	< 0.001	0.34 (0.31, 0.38)	< 0.001	0.34 (0.32, 0.36)	< 0.001	0.53 (0.47, 0.6)
45–49	< 0.001	0.59 (0.55, 0.64)	< 0.001	0.62 (0.57, 0.67)	< 0.001	0.68 (0.63, 0.74)	< 0.001	0.66 (0.63, 0.69)	0.4	0.96 (0.87, 1.06)
50–54	0.54	1.02 (0.96, 1.07)	0.4	1.03 (0.96, 1.1)	< 0.001	1.2 (1.12, 1.28)	< 0.001	1.15 (1.11, 1.2)	< 0.001	1.55 (1.44, 1.67)
55–59	< 0.001	1.49 (1.44, 1.54)	< 0.001	1.37 (1.3, 1.45)	< 0.001	1.57 (1.48, 1.66)	< 0.001	1.61 (1.55, 1.66)	< 0.001	2.13 (2.01, 2.26)
60–64	< 0.001	2.02 (1.97, 2.09)	< 0.001	1.91 (1.82, 2)	< 0.001	2.25 (2.14, 2.37)	< 0.001	2.23 (2.16, 2.29)	< 0.001	2.75 (2.61, 2.9)
65–69	< 0.001	2.51 (2.41, 2.6)	< 0.001	2.41 (2.32, 2.51)	< 0.001	2.7 (2.59, 2.82)	< 0.001	2.78 (2.71, 2.84)	< 0.001	3.13 (2.94, 3.32)
70–74	< 0.001	3.12 (2.95, 3.3)	< 0.001	3.02 (2.91, 3.13)	< 0.001	3.23 (3.11, 3.35)	< 0.001	3.38 (3.31, 3.45)	< 0.001	3.08 (2.86, 3.32)
75–79	< 0.001	3.05 (2.83, 3.29)	< 0.001	3.51 (3.4, 3.63)	< 0.001	3.66 (3.53, 3.8)	< 0.001	3.81 (3.74, 3.89)	< 0.001	2.82 (2.56, 3.1)
80–84	< 0.001	2.47 (2.24, 2.73)	< 0.001	3.69 (3.56, 3.83)	< 0.001	3.86 (3.71, 4.01)	< 0.001	4 (3.91, 4.09)	< 0.001	2.21 (1.96, 2.49)
85–89	< 0.001	2.62 (2.32, 2.96)	< 0.001	4.13 (3.96, 4.31)	< 0.001	3.94 (3.77, 4.13)	< 0.001	4.05 (3.94, 4.15)	< 0.001	1.39 (1.19, 1.61)
90–94	< 0.001	1.9 (1.63, 2.22)	< 0.001	4 (3.79, 4.22)	< 0.001	3.59 (3.38, 3.8)	< 0.001	3.53 (3.41, 3.65)	0.12	1.18 (0.96, 1.44)
95+	0.16	0.82 (0.61, 1.08)	< 0.001	2.9 (2.66, 3.15)	< 0.001	2.09 (1.89, 2.31)	< 0.001	2.42 (2.29, 2.56)	0.27	0.78 (0.5, 1.22)
Period effects										
1992–1996	< 0.001	0.44 (0.42, 0.47)	< 0.001	0.62 (0.6, 0.65)	< 0.001	0.64 (0.62, 0.66)	< 0.001	0.74 (0.72, 0.75)	< 0.001	0.71 (0.67, 0.76)
1997–2001	< 0.001	0.81 (0.78, 0.84)	< 0.001	0.8 (0.78, 0.82)	< 0.001	0.76 (0.74, 0.78)	< 0.001	0.84 (0.83, 0.86)	< 0.001	0.7 (0.67, 0.73)
2002–2006	< 0.001	0.92 (0.9, 0.93)	0.02	0.98 (0.96, 1)	< 0.001	0.93 (0.91, 0.95)	< 0.001	0.94 (0.93, 0.95)	< 0.001	0.79 (0.77, 0.82)
2007–2011	< 0.001	1.15 (1.13, 1.17)	< 0.001	1.1 (1.08, 1.12)	< 0.001	1.09 (1.07, 1.11)	< 0.001	1.05 (1.04, 1.06)	< 0.001	1.06 (1.03, 1.09)
2012–2016	< 0.001	1.46 (1.41, 1.52)	< 0.001	1.27 (1.24, 1.3)	< 0.001	1.31 (1.28, 1.34)	< 0.001	1.21 (1.19, 1.22)	< 0.001	1.33 (1.28, 1.39)
2017–2021	< 0.001	1.82 (1.71, 1.92)	< 0.001	1.47 (1.43, 1.52)	< 0.001	1.56 (1.51, 1.61)	< 0.001	1.34 (1.32, 1.37)	< 0.001	1.78 (1.67, 1.9)
Cohort effects										
1897–1901	0.25	2.67 (0.5, 14.26)	< 0.001	2.97 (2.22, 3.98)	< 0.001	3.22 (2.29, 4.54)	< 0.001	2.91 (2.48, 3.42)	0.56	1.66 (0.3, 9.15)
1902–1906	< 0.001	2.45 (1.53, 3.92)	< 0.001	3.01 (2.65, 3.41)	< 0.001	2.81 (2.44, 3.24)	< 0.001	2.74 (2.54, 2.97)	0.29	1.42 (0.74, 2.7)
1907–1911	< 0.001	1.99 (1.47, 2.71)	< 0.001	2.84 (2.6, 3.09)	< 0.001	2.65 (2.41, 2.91)	< 0.001	2.63 (2.49, 2.77)	0.09	1.4 (0.95, 2.06)
1912–1916	< 0.001	1.83 (1.42, 2.38)	< 0.001	2.38 (2.22, 2.56)	< 0.001	2.46 (2.27, 2.66)	< 0.001	2.52 (2.42, 2.63)	< 0.001	1.53 (1.14, 2.04)
1917–1921	< 0.001	1.61 (1.28, 2.02)	< 0.001	2.24 (2.09, 2.39)	< 0.001	2.18 (2.04, 2.34)	< 0.001	2.39 (2.31, 2.49)	< 0.001	1.65 (1.28, 2.13)
1922–1926	< 0.001	1.42 (1.15, 1.74)	< 0.001	2.02 (1.9, 2.14)	< 0.001	2.16 (2.02, 2.3)	< 0.001	2.21 (2.13, 2.29)	< 0.001	1.79 (1.43, 2.24)
1927–1931	0.01	1.29 (1.08, 1.55)	< 0.001	1.77 (1.67, 1.87)	< 0.001	1.9 (1.79, 2.02)	< 0.001	2.02 (1.96, 2.09)	< 0.001	1.82 (1.5, 2.22)
1932–1936	0.06	1.17 (0.99, 1.37)	< 0.001	1.56 (1.48, 1.66)	< 0.001	1.73 (1.62, 1.83)	< 0.001	1.81 (1.75, 1.87)	< 0.001	1.75 (1.47, 2.08)
1937–1941	0.47	1.05 (0.92, 1.21)	< 0.001	1.35 (1.27, 1.44)	< 0.001	1.46 (1.37, 1.56)	< 0.001	1.54 (1.48, 1.6)	< 0.001	1.6 (1.38, 1.87)
1942–1946	0.31	0.94 (0.84, 1.06)	< 0.001	1.15 (1.08, 1.23)	< 0.001	1.18 (1.1, 1.26)	< 0.001	1.28 (1.23, 1.33)	< 0.001	1.52 (1.33, 1.73)
1947–1951	< 0.001	0.87 (0.79, 0.96)	0.81	1.01 (0.94, 1.08)	0.15	0.95 (0.88, 1.02)	0.12	1.03 (0.99, 1.08)	< 0.001	1.29 (1.15, 1.44)
1952–1956	< 0.001	0.79 (0.73, 0.85)	< 0.001	0.87 (0.8, 0.94)	< 0.001	0.79 (0.72, 0.85)	< 0.001	0.85 (0.81, 0.89)	0.02	1.12 (1.02, 1.24)
1957–1961	< 0.001	0.74 (0.7, 0.78)	< 0.001	0.73 (0.67, 0.8)	< 0.001	0.65 (0.6, 0.72)	< 0.001	0.7 (0.66, 0.74)	0.26	0.95 (0.88, 1.04)
1962–1966	< 0.001	0.66 (0.63, 0.69)	< 0.001	0.61 (0.56, 0.67)	< 0.001	0.56 (0.5, 0.62)	< 0.001	0.57 (0.54, 0.61)	< 0.001	0.82 (0.76, 0.89)
1967–1971	< 0.001	0.62 (0.6, 0.64)	< 0.001	0.51 (0.46, 0.57)	< 0.001	0.5 (0.44, 0.55)	< 0.001	0.46 (0.43, 0.5)	< 0.001	0.71 (0.65, 0.78)
1972–1976	< 0.001	0.57 (0.55, 0.6)	< 0.001	0.45 (0.39, 0.51)	< 0.001	0.44 (0.38, 0.5)	< 0.001	0.38 (0.35, 0.41)	< 0.001	0.59 (0.53, 0.65)
1977–1981	< 0.001	0.56 (0.53, 0.6)	< 0.001	0.37 (0.32, 0.44)	< 0.001	0.38 (0.32, 0.45)	< 0.001	0.33 (0.3, 0.36)	< 0.001	0.52 (0.45, 0.6)
1982–1986	< 0.001	0.56 (0.51, 0.61)	< 0.001	0.33 (0.26, 0.42)	< 0.001	0.34 (0.27, 0.43)	< 0.001	0.31 (0.26, 0.35)	< 0.001	0.44 (0.36, 0.53)
1987–1991	< 0.001	0.56 (0.5, 0.63)	< 0.001	0.3 (0.2, 0.43)	< 0.001	0.3 (0.21, 0.44)	< 0.001	0.29 (0.23, 0.37)	< 0.001	0.32 (0.24, 0.43)
1992–1996	< 0.001	0.58 (0.49, 0.69)	< 0.001	0.26 (0.11, 0.59)	< 0.001	0.28 (0.12, 0.64)	< 0.001	0.27 (0.16, 0.45)	< 0.001	0.25 (0.13, 0.46)

Categories	Deaths									
	China		France		England		United States of America		Russian Federation	
	*p*	RR (95% CI)	*p*	RR (95% CI)	*p*	RR (95% CI)	*p*	RR (95% CI)	*p*	RR (95% CI)
Age effects										
25–29	< 0.001	0.12 (0.11, 0.14)	< 0.001	0.03 (0.02, 0.05)	< 0.001	0.02 (0.01, 0.04)	< 0.001	0.02 (0.02, 0.03)	< 0.001	0.07 (0.05, 0.1)
30–34	< 0.001	0.14 (0.12, 0.15)	< 0.001	0.06 (0.05, 0.08)	< 0.001	0.06 (0.04, 0.08)	< 0.001	0.05 (0.05, 0.06)	< 0.001	0.12 (0.09, 0.14)
35–39	< 0.001	0.22 (0.2, 0.25)	< 0.001	0.13 (0.1, 0.15)	< 0.001	0.12 (0.09, 0.15)	< 0.001	0.13 (0.11, 0.14)	< 0.001	0.22 (0.19, 0.25)
40–44	< 0.001	0.35 (0.33, 0.38)	< 0.001	0.25 (0.21, 0.28)	< 0.001	0.24 (0.2, 0.29)	< 0.001	0.28 (0.26, 0.3)	< 0.001	0.43 (0.39, 0.48)
45–49	< 0.001	0.49 (0.46, 0.52)	< 0.001	0.45 (0.4, 0.5)	< 0.001	0.49 (0.42, 0.56)	< 0.001	0.55 (0.52, 0.58)	< 0.001	0.78 (0.71, 0.85)
50–54	< 0.001	0.84 (0.8, 0.87)	< 0.001	0.72 (0.66, 0.79)	< 0.001	0.84 (0.75, 0.94)	0.03	0.95 (0.91, 1)	< 0.001	1.24 (1.16, 1.34)
55–59	< 0.001	1.26 (1.22, 1.31)	0.27	1.05 (0.97, 1.13)	< 0.001	1.26 (1.15, 1.38)	< 0.001	1.41 (1.35, 1.46)	< 0.001	1.76 (1.66, 1.87)
60–64	< 0.001	1.76 (1.71, 1.81)	< 0.001	1.58 (1.48, 1.69)	< 0.001	1.88 (1.74, 2.03)	< 0.001	2.02 (1.96, 2.09)	< 0.001	2.37 (2.25, 2.51)
65–69	< 0.001	2.29 (2.21, 2.37)	< 0.001	2.2 (2.08, 2.32)	< 0.001	2.55 (2.39, 2.72)	< 0.001	2.64 (2.57, 2.71)	< 0.001	2.88 (2.73, 3.05)
70–74	< 0.001	3.03 (2.9, 3.17)	< 0.001	3.04 (2.91, 3.18)	< 0.001	3.31 (3.14, 3.49)	< 0.001	3.42 (3.34, 3.5)	< 0.001	3.06 (2.87, 3.26)
75–79	< 0.001	3.23 (3.04, 3.42)	< 0.001	3.96 (3.79, 4.13)	< 0.001	4.06 (3.87, 4.26)	< 0.001	4.12 (4.04, 4.21)	< 0.001	3.02 (2.8, 3.26)
80–84	< 0.001	2.9 (2.69, 3.12)	< 0.001	4.84 (4.62, 5.07)	< 0.001	4.87 (4.63, 5.12)	< 0.001	4.73 (4.62, 4.84)	< 0.001	2.62 (2.38, 2.87)
85–89	< 0.001	3.32 (3.03, 3.63)	< 0.001	6.08 (5.76, 6.43)	< 0.001	5.58 (5.26, 5.93)	< 0.001	5.15 (5.02, 5.29)	< 0.001	1.78 (1.57, 2.01)
90–94	< 0.001	2.62 (2.33, 2.95)	< 0.001	6.58 (6.14, 7.04)	< 0.001	5.55 (5.15, 5.98)	< 0.001	4.9 (4.74, 5.07)	< 0.001	1.67 (1.41, 1.97)
95+	< 0.001	1.49 (1.2, 1.85)	< 0.001	6.49 (5.92, 7.12)	< 0.001	5.34 (4.81, 5.93)	< 0.001	3.9 (3.71, 4.11)	0.02	1.51 (1.08, 2.12)
Period effects										
1992–1996	< 0.001	0.48 (0.45, 0.5)	< 0.001	0.69 (0.66, 0.72)	< 0.001	0.71 (0.67, 0.74)	< 0.001	0.75 (0.74, 0.77)	< 0.001	0.73 (0.69, 0.77)
1997–2001	< 0.001	0.86 (0.83, 0.89)	< 0.001	0.84 (0.81, 0.86)	< 0.001	0.79 (0.76, 0.82)	< 0.001	0.85 (0.84, 0.86)	< 0.001	0.73 (0.7, 0.76)
2002–2006	< 0.001	0.94 (0.92, 0.96)	0.18	0.98 (0.96, 1.01)	< 0.001	0.9 (0.88, 0.93)	< 0.001	0.94 (0.93, 0.95)	< 0.001	0.81 (0.79, 0.84)
2007–2011	< 0.001	1.12 (1.1, 1.14)	< 0.001	1.05 (1.03, 1.08)	0.14	1.02 (0.99, 1.05)	< 0.001	1.02 (1.01, 1.03)	< 0.001	1.06 (1.03, 1.09)
2012–2016	< 0.001	1.38 (1.34, 1.42)	< 0.001	1.2 (1.16, 1.24)	< 0.001	1.26 (1.21, 1.3)	< 0.001	1.19 (1.18, 1.21)	< 0.001	1.3 (1.25, 1.35)
2017–2021	< 0.001	1.68 (1.61, 1.75)	< 0.001	1.4 (1.34, 1.46)	< 0.001	1.55 (1.48, 1.62)	< 0.001	1.37 (1.34, 1.4)	< 0.001	1.68 (1.59, 1.77)
Cohort effects										
1897–1901	0.06	3.12 (0.95, 10.22)	< 0.001	3.06 (2.39, 3.93)	< 0.001	3.44 (2.57, 4.61)	< 0.001	3.15 (2.76, 3.59)	0.29	1.93 (0.57, 6.49)
1902–1906	< 0.001	2.44 (1.67, 3.58)	< 0.001	3.36 (2.95, 3.83)	< 0.001	3.16 (2.7, 3.69)	< 0.001	3.03 (2.83, 3.25)	0.04	1.69 (1.02, 2.81)
1907–1911	< 0.001	2.14 (1.7, 2.69)	< 0.001	3.22 (2.91, 3.55)	< 0.001	2.99 (2.67, 3.35)	< 0.001	2.91 (2.76, 3.06)	0.01	1.53 (1.12, 2.08)
1912–1916	< 0.001	1.98 (1.63, 2.39)	< 0.001	2.75 (2.52, 3.01)	< 0.001	2.84 (2.58, 3.12)	< 0.001	2.8 (2.68, 2.92)	< 0.001	1.62 (1.3, 2.03)
1917–1921	< 0.001	1.75 (1.48, 2.07)	< 0.001	2.62 (2.41, 2.85)	< 0.001	2.56 (2.35, 2.79)	< 0.001	2.65 (2.55, 2.75)	< 0.001	1.76 (1.45, 2.14)
1922–1926	< 0.001	1.55 (1.33, 1.8)	< 0.001	2.38 (2.2, 2.57)	< 0.001	2.54 (2.34, 2.76)	< 0.001	2.43 (2.34, 2.52)	< 0.001	1.92 (1.62, 2.27)
1927–1931	< 0.001	1.41 (1.23, 1.61)	< 0.001	2.09 (1.93, 2.26)	< 0.001	2.2 (2.03, 2.39)	< 0.001	2.2 (2.13, 2.29)	< 0.001	1.94 (1.67, 2.25)
1932–1936	< 0.001	1.27 (1.13, 1.42)	< 0.001	1.82 (1.68, 1.98)	< 0.001	1.98 (1.81, 2.16)	< 0.001	1.94 (1.87, 2.02)	< 0.001	1.85 (1.62, 2.12)
1937–1941	0.01	1.14 (1.03, 1.26)	< 0.001	1.56 (1.43, 1.71)	< 0.001	1.64 (1.49, 1.8)	< 0.001	1.62 (1.56, 1.69)	< 0.001	1.68 (1.49, 1.9)
1942–1946	0.9	1.01 (0.92, 1.1)	< 0.001	1.3 (1.18, 1.44)	< 0.001	1.28 (1.15, 1.42)	< 0.001	1.32 (1.26, 1.38)	< 0.001	1.58 (1.42, 1.76)
1947–1951	0.01	0.91 (0.85, 0.98)	0.07	1.11 (0.99, 1.24)	0.92	0.99 (0.88, 1.12)	0.09	1.04 (0.99, 1.1)	< 0.001	1.32 (1.19, 1.46)
1952–1956	< 0.001	0.81 (0.76, 0.86)	0.16	0.92 (0.81, 1.04)	< 0.001	0.79 (0.69, 0.91)	< 0.001	0.84 (0.8, 0.89)	0.01	1.13 (1.03, 1.24)
1957–1961	< 0.001	0.74 (0.7, 0.77)	< 0.001	0.74 (0.65, 0.85)	< 0.001	0.64 (0.55, 0.74)	< 0.001	0.68 (0.64, 0.72)	0.15	0.94 (0.85, 1.02)
1962–1966	< 0.001	0.64 (0.61, 0.66)	< 0.001	0.59 (0.51, 0.69)	< 0.001	0.52 (0.44, 0.62)	< 0.001	0.55 (0.51, 0.58)	< 0.001	0.79 (0.72, 0.87)
1967–1971	< 0.001	0.59 (0.56, 0.61)	< 0.001	0.48 (0.4, 0.57)	< 0.001	0.45 (0.37, 0.54)	< 0.001	0.43 (0.4, 0.47)	< 0.001	0.66 (0.59, 0.74)
1972–1976	< 0.001	0.53 (0.5, 0.55)	< 0.001	0.4 (0.32, 0.49)	< 0.001	0.38 (0.31, 0.48)	< 0.001	0.35 (0.32, 0.39)	< 0.001	0.54 (0.47, 0.61)
1977–1981	< 0.001	0.5 (0.47, 0.54)	< 0.001	0.31 (0.23, 0.41)	< 0.001	0.31 (0.23, 0.42)	< 0.001	0.3 (0.26, 0.34)	< 0.001	0.47 (0.4, 0.56)
1982–1986	< 0.001	0.48 (0.44, 0.53)	< 0.001	0.25 (0.17, 0.38)	< 0.001	0.28 (0.19, 0.43)	< 0.001	0.27 (0.22, 0.32)	< 0.001	0.39 (0.31, 0.48)
1987–1991	< 0.001	0.47 (0.42, 0.53)	< 0.001	0.21 (0.11, 0.39)	< 0.001	0.23 (0.12, 0.44)	< 0.001	0.25 (0.19, 0.33)	< 0.001	0.28 (0.2, 0.4)
1992–1996	< 0.001	0.47 (0.39, 0.56)	0.01	0.16 (0.04, 0.66)	0.03	0.2 (0.05, 0.83)	< 0.001	0.22 (0.12, 0.41)	< 0.001	0.19 (0.09, 0.43)

In China, the incidence of MM was closely related to age, with 70–74 (3.12, 95% CI: 2.95–3.30) and 75–79 (3.05, 95% CI: 2.83–3.29) being the age groups with the highest RR of incidence. For deaths, 70–74 (3.03, 95% CI: 2.90–3.17), 75–79 (3.23, 95% CI: 3.04–3.42), and 85–89 (3.32, 95% CI: 3.03–3.63) years showed the highest RR. For the period effect, the RR for incidence ranged from 0.44 (95% CI. 0.42–0.47) to 1.82 (95% CI: 1.71–1.92) and the RR for deaths increased from 0.48 (95% CI: 0.45–0.50) to 1.68 (95% CI: 1.61–1.75). In terms of cohort effects, the RR for incidence in 1996 was 0.58 (95% CI: 0.49–0.69) and the RR for deaths was 0.47 (95% CI: 0.39–0.56).

In the United States of America, the incidence of MM was most significant between 80 and 84 years (4.00, 95% CI: 3.91–4.09) and 85–89 years (4.05, 95% CI: 3.94–4.15). The RR for deaths was highest in the 85–89 years (5.15, 95% CI: 5.02–5.29) group. For period effects, the RR for incidence increased from 0.74 (95% CI: 0.72–0.75) to 1.34 (95% CI: 1.32–1.37), and for deaths from 0.75 (95% CI: 0.74–0.77) to 1.37 (95% CI: 1.34–1.40). The RRs for incidence and deaths for the 1996 cohort were 0.27 (95% CI: 0.16–0.45) and 0.22 (95% CI: 0.12–0.41), respectively.

In the Russian Federation, the incidence of MM was highest in the 65–69 (3.13, 95% CI: 2.94–3.32) and 70–74 (3.08, 95% CI: 2.86–3.32) age groups, while the 70–74 (3.06, 95% CI: 2.87–3.26) and 75–79 (3.02, 95% CI: 2.80–3.26) were the groups with the highest RR for deaths. Period effects showed that the RR for incidence increased from 0.71 (95% CI: 0.67–0.76) to 1.78 (95% CI: 1.67–1.90), and the RR for deaths rose from 0.73 (95% CI: 0.69–0.77) to 1.68 (95% CI: 1.59–1.77). The RRs for incidence and deaths for the 1996 cohort were 0.25 (95% CI: 0.13–0.46) and 0.19 (95% CI: 0.09–0.43), respectively.

In England, incidence was highest in the 75–79 (3.66, 95% CI: 3.53–3.80), 80–84 (3.86, 95% CI: 3.71–4.01) and 85–89 (3.94, 95% CI: 3.77–4.13) age groups. Deaths, on the other hand, were most prominent in the 85–89 (5.58, 95% CI: 5.26–5.93), 90–94 (5.55, 95% CI: 5.15–5.98) and 95+ (5.34, 95% CI: 4.81–5.93) age groups. The RR for incidence increased from 0.64 (95% CI: 0.62–0.66) to 1.56 (95% CI: 1.51–1.61), and the RR for deaths increased from 0.71 (95% CI: 0.67–0.74) to 1.55 (95% CI: 1.48–1.62) during the period effect. For the 1996 cohort, the RRs for incidence and deaths were 0.28 (95% CI: 0.12–0.64) and 0.20 (95% CI: 0.05–0.83), respectively.

In France, 85–89 (4.13, 95% CI: 3.96–4.31) and 90–94 (4.00, 95% CI: 3.79–4.22) years were the age groups with the highest RR of incidence. Deaths were highest in the 85–89 (6.08, 95% CI: 5.76–6.43), 90–94 (6.58, 95% CI: 6.14–7.04) and 95+ (6.49, 95% CI: 5.92–7.12) age groups. Period effects showed an increase in the RR for incidence from 0.62 (95% CI: 0.60–0.65) to 1.47 (95% CI: 1.43–1.52) and for deaths from 0.69 (95% CI: 0.66–0.72) to 1.40 (95% CI: 1.34–1.46). The RRs for incidence and deaths for the 1996 cohort were 0.26 (95% CI: 0.11–0.59) and 0.16 (95% CI: 0.04–0.66), respectively.

### 3.4 Auto Regressive Integrated Moving Average Model Analysis

**FIGURE 6:**
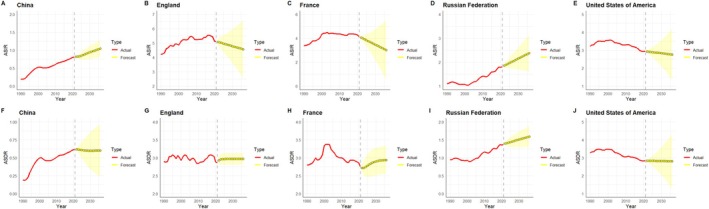
Trends and projections of ASIR and ASDR for multiple myeloma in the five countries for the period 1990–2036. The yellow region in the figures shows the upper and lower limits of the 95% uncertainty intervals (95% UI). ASIR, age‐standardised incidence rates; ASDR, age‐standardised death rates.

Figure 6 illustrates the projected trends in ASIR and ASDR for MM in the five countries between 2022 and 2036. During this period, the ASIR of MM in China and the Russian Federation shows an increasing trend, and the ASDR remains stable, while the ASIR and ASDR of MM in England, France and the United States of America tend to be stable overall.

## 4 Discussion

Our study shows different trends in MM incidence and deaths in the five countries. China and the Russian Federation showed a sustained increase in the burden of MM, particularly in the older age groups; the United States of America showed a decreasing trend, indicating a reduced burden of disease; and England and France showed an overall increase in incidence but a relatively stable death rate. Age effects were evident across countries, with the heaviest burden in the older age groups, while period and cohort effects varied significantly across countries.

China has made significant progress in medical technology and disease screening in recent years, and the incidence of MM has continued to rise over the past few decades, with a significant increase especially in the older age groups, a trend that may be closely related to changes in the ageing population or due to a general increase in unhealthy lifestyles, such as smoking, drinking, sedentary lifestyle, physical inactivity, and overweight [24, 25, 26]. Meanwhile, the trend of ageing is more pronounced in developed provinces with a higher proportion of elderly people, further contributing to the increase in MM incidence and death rates [27]. In addition, despite improvements in health insurance, health insurance coverage and quality of services in less developed and rural areas are still low, and many patients are unable to receive timely diagnosis and treatment due to excessive financial burden [28]. According to the statistics of CONCORD‐32018, the overall 5‐year survival rate of MM in China is 24.8%, which is still significantly lower than that of developed countries such as Europe and the United States, which is 46.7% [29].

In contrast, there has been a notable increase in morbidity and mortality rates in the Russian Federation over the past three decades. This phenomenon is closely linked to the country's persistent environmental pollution [30]. The relative scarcity of medical resources in the Russian Federation, particularly in rural regions, coupled with elevated rates of tobacco consumption, is a probable contributor to the observed rise in MM incidence and mortality [31, 32]. It has been suggested that smoking may be associated with an increased risk of MM and that healthcare disparities may result in patients being diagnosed later and receiving inadequate treatment, thus affecting their prognosis [33, 34].

In the United States of America, by contrast, the incidence and death rates are on a downward trend, although ageing and some unhealthy lifestyle factors continue to exert some pressure on incidence and death in some groups. The United States of America has made relatively rapid advances in medical technology, disease screening, and early diagnostic techniques, which have greatly improved the early detection and treatment of MM, thereby increasing patient survival rates [35, 36, 37]. Its 5‐year productivity has increased from 35% in 2000 to over 60% in 2020 [38]. In addition, efforts to control lifestyle factors such as obesity and smoking in the United States of America in recent years may also have played a positive role in slowing the increase in incidence rates [39, 40].

In England and France, the incidence of MM has risen, particularly in older age groups, despite long‐term progress in cancer treatment in both countries [41]. Studies in France and England have shown that declining immune system function in older age groups leads to weakened resilience in response to haematological disorders such as MM, further contributing to the increased incidence and deaths of MM [42]; high‐quality healthcare and access to medicines can reduce cancer deaths [43]. Meanwhile, although the health insurance systems in both countries are relatively well‐developed, the use of healthcare resources is still somewhat limited among some low‐income groups, which may affect the timely treatment of MM and the prognosis of patients [44, 45].

Our report also shows that men have higher ASIR and ASDR than women. Some known causative factors, such as smoking and obesity, are associated with higher deaths in MM [25]. Physiological differences such as a greater tendency to smoke in the male population and the fact that men's immune systems are generally weaker in response to disease, with weaker immunoregulation relative to women, may make men more susceptible to MM [46, 47]. The combination of these factors explains why the male population exhibits a higher burden of incidence and deaths in MM.

Age has a significant impact on MM incidence and deaths. The presence of a well‐established risk factor has been consistently demonstrated by research, which indicates that there is a notable increase in the incidence of myeloma as the age of the population concerned increases [48, 49]. Our study shows that incidence and deaths are higher among older people in the five countries. That is, the burden of MM is concentrated in the elderly. This result may be due to many factors. First, with age, the function of the human immune system gradually declines, and immune surveillance capacity is weakened, making the elderly more susceptible to malignant tumours, including MM [50]. Second, with age, multiple chronic diseases, such as cardiovascular disease, diabetes mellitus, and renal failure, are prevalent in the elderly population, and these comorbidities not only increase the risk of MM but also increase the difficulty and complexity of treatment, resulting in poorer outcomes [2]. For example, cardiovascular disease may limit the intensity of treatment that patients can tolerate [51]. In addition, elderly patients may be less fit and tolerant, leading to more side effects or complications during treatment, which may affect the effectiveness of the treatment and increase the risk of deaths [52]. These factors therefore suggest that age is an important risk factor as a risk factor for MM that cannot be ignored, and that disease management, especially in the elderly population, requires more refined and individualised intervention strategies.

At the same time, the period effect suggests that prevalence rates have continued to rise over time in the five countries. This trend may be closely related to the continuous improvement of early diagnostic techniques and screening tools, increased environmental pollution due to industrialisation, and the prevalence of unhealthy lifestyles [5, 25, 53]. At the same time, death rates in these countries have continued to rise, a trend that may be closely related to a number of factors [54]. For example, late diagnosis is a key factor, as many patients are diagnosed with MM when the disease has progressed to a more advanced stage due to the lack of early symptoms, limiting the effectiveness of treatment, as well as the increasing age of the patient and common comorbidities (e.g., cardiovascular disease and renal failure) further increasing the difficulty in treating the disease and exacerbating the risk of death, and the inability of patients in developing countries and in low‐income areas to undergo early autologous treatment, which increases the risk of disease recurrence.

In addition, in five countries, cohort effects showed that early birth cohorts tended to experience higher MM incidence and deaths. This trend may be closely related to historically important societal events, particularly radiation exposure during the World Wars and increased environmental pollution in the post‐war period, including contamination of food and water sources. These factors may have increased the risk of MM and the burden of disease in the early birth cohort [53, 55]. Notably, epidemiological evidence from large cohort studies suggests that ionising radiation may be associated with an increased risk of MM, indicating that environmental exposures experienced by early birth cohorts may contribute to their overall disease burden [56, 57, 58]. With the rapid advancement of information technology and the popularity of the Internet, the public's access to health information has gradually become more diversified [59]. In the five countries, especially in younger age groups, online platforms provide easy access to scientific information on disease prevention and health management that can help to reduce environmental and lifestyle‐related risk factors, potentially reducing the incidence of MM in late‐born cohorts [60, 61]. At the same time, the burden of disease has been reduced to a certain extent with the advancement of health education and the deepening of the community's awareness of the impact of the environment and lifestyle [62].

ASIR is projected to continue to rise in China and the Russian Federation over the next 14 years, which is closely related to increasing ageing, lifestyle changes, and environmental factors in both countries. In contrast, the incidence of MM in the United States of America, England, and France is projected to be on a downward trend, reflecting advances in public health policies, early screening, and treatment technologies in these countries. However, despite significant changes in the ASIR for MM across countries, the ASDR for each country is expected to remain stable. Due to the chronic nature of MM, patients are usually able to live with the disease for a longer period of time, and while advances in treatments have improved the quality of survival, limitations in treatments and the difficulty of managing high‐risk groups have prevented significant reductions in death rates [54].

Nevertheless, there are several noteworthy limitations to this study. Firstly, the availability and quality of raw data is a key limitation in estimating MM incidence and deaths. As some national or regional healthcare systems are unable to provide high‐quality or sufficient MM data, this has led to variations in the estimation of MM incidence and deaths in the GBD model. Secondly, the failure of this study to analyse differences at the local level at a more granular level is also a limitation, given the differences in health problems, access to healthcare services, and resource allocation in different regions. Thirdly, the present study was impeded by the fact that the GBD 2021 database, which was utilised for this study, did not provide state‐level morbidity or mortality data stratified by race. This precluded the possibility of conducting a systematic race‐level analysis at the time, although this is a potential avenue for future research. Finally, this study was based primarily on GBD 2021 cross‐sectional data between 1992 and 2021, focusing on age, period, and cohort effects. However, future cohort studies in different countries will continue to be essential to assess relative risk in specific areas and time periods, as well as differences in risk between different groups.

## 5 Conclusions

The present study analysed trends in MM incidence and deaths in five countries that are permanent members of the United Nations Security Council. We found that the burden of MM continues to rise in China and the Russian Federation, especially in older age groups, while the United States of America, England, and France show a decreasing trend in incidence and a stable death rate. The study also revealed significant differences in MM incidence and deaths and the social, environmental, and medical factors behind them in different countries. These findings suggest that countries should pay more attention to the prevention and treatment of MM in the elderly population, especially to promote the advancement of early screening and treatment methods, as well as to strengthen interventions against unhealthy lifestyles and environmental pollution. Future studies should delve deeper into the risk of MM in different regions and groups to provide data support for the development of more precise public health policies.

## Author Contributions

**Huiqiang Wu:** writing – original draft, conceptualization. **Zhiyin Cai:** conceptualization, writing – original draft. **Wanyi Liu:** conceptualization, methodology. **Zechuan Wang:** conceptualization, software. **Baoying Qiu:** data curation. **Weihui Liu:** formal analysis, resources. **Weihuang Zhuang:** writing – review and editing.

## Ethics Statement

The authors have nothing to report.

## Consent

The authors have nothing to report.

## Conflicts of Interest

The authors declare no conflicts of interest.

## Data Availability

The authors have nothing to report.
